# Supplementary material for: Spontaneous skyrmionic lattice from anisotropic symmetric exchange in a Ni-halide monolayer
Source: Nat Commun. 2020 Nov 13;11:5784. doi: 10.1038/s41467-020-19535-w (PMC7666143; doi:10.1038/s41467-020-19535-w)
Supplement: Supplementary file 2 — Supplementary Information [file 41467_2020_19535_MOESM2_ESM.pdf]

Supplementary Information to Spontaneous skyrmionic lattice from anisotropic symmetric exchange in a Ni-halide monolayer

Danila Amoroso,[1] Paolo Barone,[1] and Silvia Picozzi[1]

[1]National Research Council CNR-SPIN, c/o Università degli Studi G. DAnnunzio, I-66100 Chieti, Italy

SI. THE MAGNETIC EXCHANGE TENSOR IN NIX$_2$ MONOLAYERS

The exchange-coupling tensor considered in the main text (Table I) for a Ni-Ni pair whose bonding vector is chosen parallel to the cartesian axis x (denoted as the Ni$_0$-Ni$_1$ magnetic pair in Fig. S1) is

$$\mathbf{J}^{1\hat{R}(0^\circ)} = \begin{pmatrix} J_{xx} & 0 & 0 \\ 0 & J_{yy} & J_{yz} \\ 0 & J_{yz} & J_{zz} \end{pmatrix} \tag{S1}$$

The corresponding tensor for the symmetry-equivalent magnetic pairs rotated by $\pm 120°$, *i.e.* Ni$_0$-Ni$_3$ and Ni$_0$-Ni$_5$ pairs shown in Fig. S1, can be deduced via the three-fold rotational symmetry operation belonging to the D_{3d} point group of the considered NiX$_2$ monolayers, reading:

$$\mathbf{J}^{1\hat{R}(\mp 120^\circ)} = \begin{pmatrix} \frac{1}{4}(J_{xx}+3J_{yy}) & \pm\frac{\sqrt{3}}{4}(J_{xx}-J_{yy}) & \pm\frac{\sqrt{3}}{2}J_{yz} \\ \pm\frac{\sqrt{3}}{4}(J_{xx}-J_{yy}) & \frac{1}{4}(3J_{xx}+J_{yy}) & -\frac{1}{2}J_{yz} \\ \pm\frac{\sqrt{3}}{2}J_{yz} & -\frac{1}{2}J_{yz} & J_{zz} \end{pmatrix} \tag{S2}$$

The tensor (S1) can be diagonalized analytically, with eigenvalues (λ) and eigenvectors ($\boldsymbol{\nu}$) given by

$$\begin{cases} \lambda_\alpha = \frac{1}{2}\left(J_{yy}+J_{zz}-\sqrt{4J_{yz}^2+(J_{yy}-J_{zz})^2}\right) \\ \lambda_\beta = J_{xx} \\ \lambda_\gamma = \frac{1}{2}\left(J_{yy}+J_{zz}+\sqrt{4J_{yz}^2+(J_{yy}-J_{zz})^2}\right) \end{cases}, \quad \begin{cases} \boldsymbol{\nu}_\alpha = \left(0, -\mathrm{sgn}(J_{yz})\sqrt{\frac{1-\cos\theta}{2}}, \sqrt{\frac{1+\cos\theta}{2}}\right) \\ \boldsymbol{\nu}_\beta = (1,0,0) \\ \boldsymbol{\nu}_\gamma = \left(0, \sqrt{\frac{1+\cos\theta}{2}}, \mathrm{sgn}(J_{yz})\sqrt{\frac{1-\cos\theta}{2}}\right) \end{cases} \tag{S3}$$

where $\cos\theta = \frac{J_{yy}-J_{zz}}{\sqrt{4J_{yz}^2+(J_{yy}-J_{zz})^2}}$.

From the analytic expression of the eigenvectors, it is clear that the off-diagonal terms of the exchange, i.e. the J_{yz} for the Ni-Ni bond parallel to the x-axis, are a direct measure of the deviation from coplanarity, inducing a

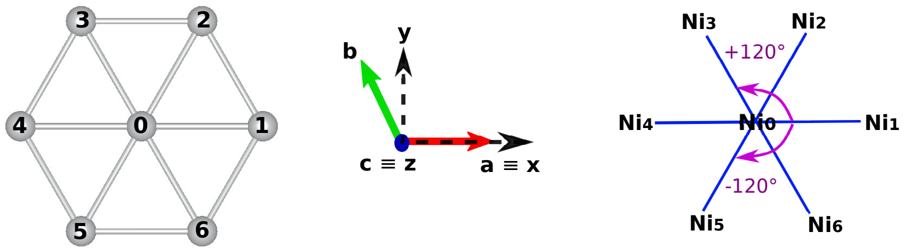

FIG. S1: Schematic representation of the six nearest neighbours surrounding a selected central magnetic site (Ni$_0$). Ni atoms are arranged in a triangular lattice, with nearest-neighbour sites labeled counter-clockwise. Components of the **J** exchange tensor reported in the main text (Table I) were calculated for the Ni$_0$-Ni$_1$ pair, whose bonding vector is parallel to the cartesian axis x, as shown in the central panel; corresponding components for the other symmetry-equivalent pairs in the chosen cartesian reference system can be deduced via the three-fold rotoinversion symmetry associated to the D_{3d} point group of the analysed NiX$_2$ monolayers. The cartesian components of the exchange tensor for Ni$_0$-Ni$_3$ and Ni$_0$-Ni$_5$ bonds are therefore obtained via a rotation of the local cartesian reference system (with axis x parallel to the chosen bond) by $\pm 120°$ around the c axis, opposite pairs being related by inversion symmetry. The same symmetry arguments also apply for the second- and third-nearest neighbour interactions [shown in Fig. S2(c-e)].

canting of the two-site anisotropy axes from the perpendicular z direction. This can be even clearer by considering two extreme cases, that is either $J_{yz} = 0$ or $(J_{yy} - J_{zz}) = 0$ - *i.e.* isotropic diagonal terms $J_{xx} = J_{yy} = J_{zz} = J$ - in the above expressions :

1) $J_{yz} = 0 \rightarrow cos\theta = 1$

$$\begin{cases} \lambda_\alpha = J_{zz} \\ \lambda_\beta = J_{xx} \\ \lambda_\gamma = J_{yy} \end{cases} \qquad \begin{cases} \boldsymbol{\nu}_\alpha = (0,0,1) \\ \boldsymbol{\nu}_\beta = (1,0,0) \\ \boldsymbol{\nu}_\gamma = (0,1,0) \end{cases}$$

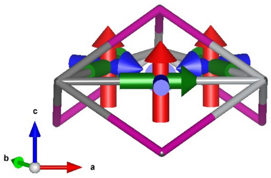

implying coplanar principal axes on the Ni triangular lattice and locally parallel to the x, y, z cartesian axes, i.e. spins interaction independent on the Ni-I-Ni-I plaquettes, as shown in the inset ($\boldsymbol{\nu}_\alpha$-red, $\boldsymbol{\nu}_\beta$-green, $\boldsymbol{\nu}_\gamma$-blue). In this case, there are no terms able to drive the formation of a non-coplanar spin-texture, which can then result into a trivial non-collinear spin-configuration, still driven by the magnetic frustration, as it results from performed MC simulations test.

2) $(J_{yy} - J_{zz}) = 0 \rightarrow cos\theta = 0$; $J_{xx} = J_{yy} = J_{zz} = J$

$$\begin{cases} \lambda_\alpha = J - J_{yz} \\ \lambda_\beta = J \\ \lambda_\gamma = J + J_{yz} \end{cases} \qquad \begin{cases} \boldsymbol{\nu}_\alpha = \left(0, -\mathrm{sgn}(J_{yz})\sqrt{\frac{1}{2}}, \sqrt{\frac{1}{2}}\right) \\ \boldsymbol{\nu}_\beta = (1,0,0) \\ \boldsymbol{\nu}_\gamma = \left(0, \sqrt{\frac{1}{2}}, \mathrm{sgn}(J_{yz})\sqrt{\frac{1}{2}}\right) \end{cases}$$

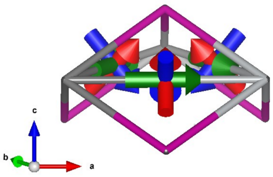

which still introduces non-coplanarity of principal axis, as shown in the inset; nevertheless, having reduced the strenght of the global anisotropy in terms of energy, the formation of the non-coplanar spin-texture, i.e. the A2Sk, would depend on the competition between the isotropic term and the anisotropic J_{yz}. In fact, from MC simulations test (not reported here) in this extreme case an increased value of J_{yz} is required to obtain the spontaneous formation of the A2Sk-lattice.

Moreover, the sign of the off-diagonal J_{yz} component, not affecting the principal values that depend on J_{yz}^2, determines the direction of the principal axes, being responsible for the change of sign of eigenvectors components (S3) and, consequently, for the change of chirality discussed in the main text and showed in Fig. S4.

In the basis of the principal axes, the exchange tensor for a given spin pair is diagonal:

$$\boldsymbol{J}_1^{diag} = \begin{pmatrix} \lambda_\alpha & 0 & 0 \\ 0 & \lambda_\beta & 0 \\ 0 & 0 & \lambda_\gamma \end{pmatrix} \tag{S4}$$

The nearest-neighbour exchange-coupling Hamiltonian can be written in such basis as:

$$H = \frac{1}{2}\sum_{i\neq j}\left(\lambda_\alpha S_i^\alpha S_j^\alpha + \lambda_\beta S_i^\beta S_j^\beta + \lambda_\gamma S_i^\gamma S_j^\gamma\right) \tag{S5}$$

where the local orthogonal basis $\{\alpha\beta\gamma\}$ depends on each spin pair; being the principal axes basis orthogonal and assuming $\lambda_\alpha = \lambda_\beta$ (in agreement with the estimated values reported in Table I of the main text, and neglecting the small difference between λ_α and λ_β found for NiI_2), the Hamiltonian can be equivalently expressed as

$$H = \frac{1}{2}\sum_{i\neq j}\left(J'\mathbf{S}_i \cdot \mathbf{S}_j + K S_i^\gamma S_j^\gamma\right) \tag{S6}$$

where $J' = (\lambda_\alpha + \lambda_\beta)/2$ can be seen as an isotropic exchange parameter and $K = (\lambda_\gamma - J')$ parametrizes the Kitaev-like anisotropic exchange interaction coefficient, as in Refs [1, 2]. Using the estimated values given in Table I of the main text, numerical values are $J' = -5.10, -6.0, -8.1$ meV and $K = 0.0, 0.3, 3.3$ meV for $NiCl_2$, $NiBr_2$ and NiI_2 monolayers, respectively. Such decomposition further confirms that the anisotropic contribution is not negligible in $NiBr_2$ and NiI_2, being far more pronounced in the latter system.

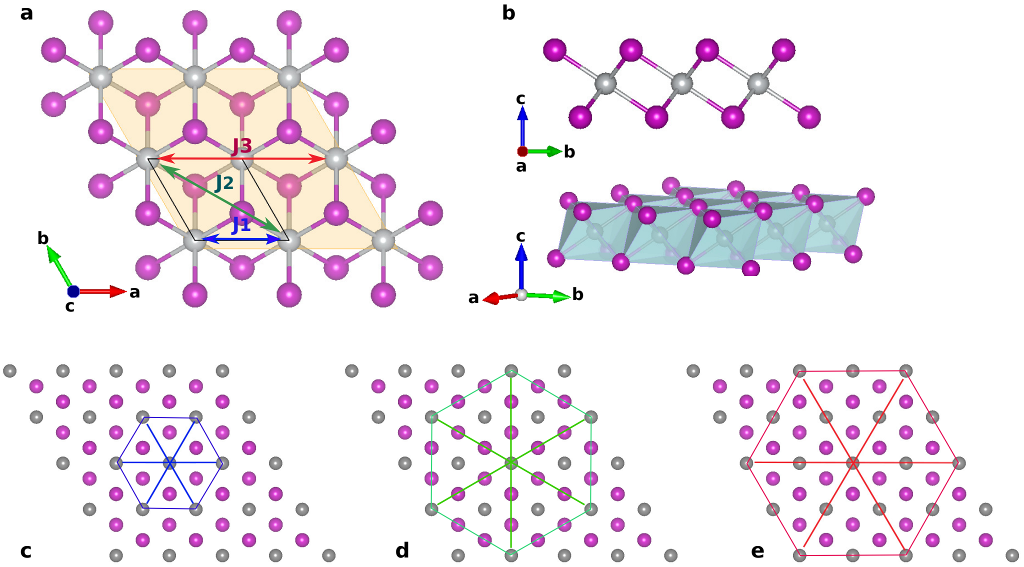

FIG. S2: Crystal structure of nickel dihalides (NiX_2) monolayers; halogen atom (X) is in purple color. (a) Top view showing the considered first-, second- and third-nearest neighbour magnetic interactions labeled as J_1, J_2 and J_3, respectively. Half-light $\{a, b\}$-plane containing Ni atoms aims at visualizing the relative position of the X-atoms with respect to the Ni layer (below and above along the perpendicular c direction). (b) Side view highlighting Ni-X bonds and the NiX_6 edge-shared octahedra. (c, d, e) Schematic representation of the six first-, second- and third-nearest neighbour Ni atoms; all are related by three-fold rotoinversion symmetry.

SII. THE TWO-SITE ANISOTROPY AS CHIRAL INTERACTION IN MONOLAYER NII$_2$

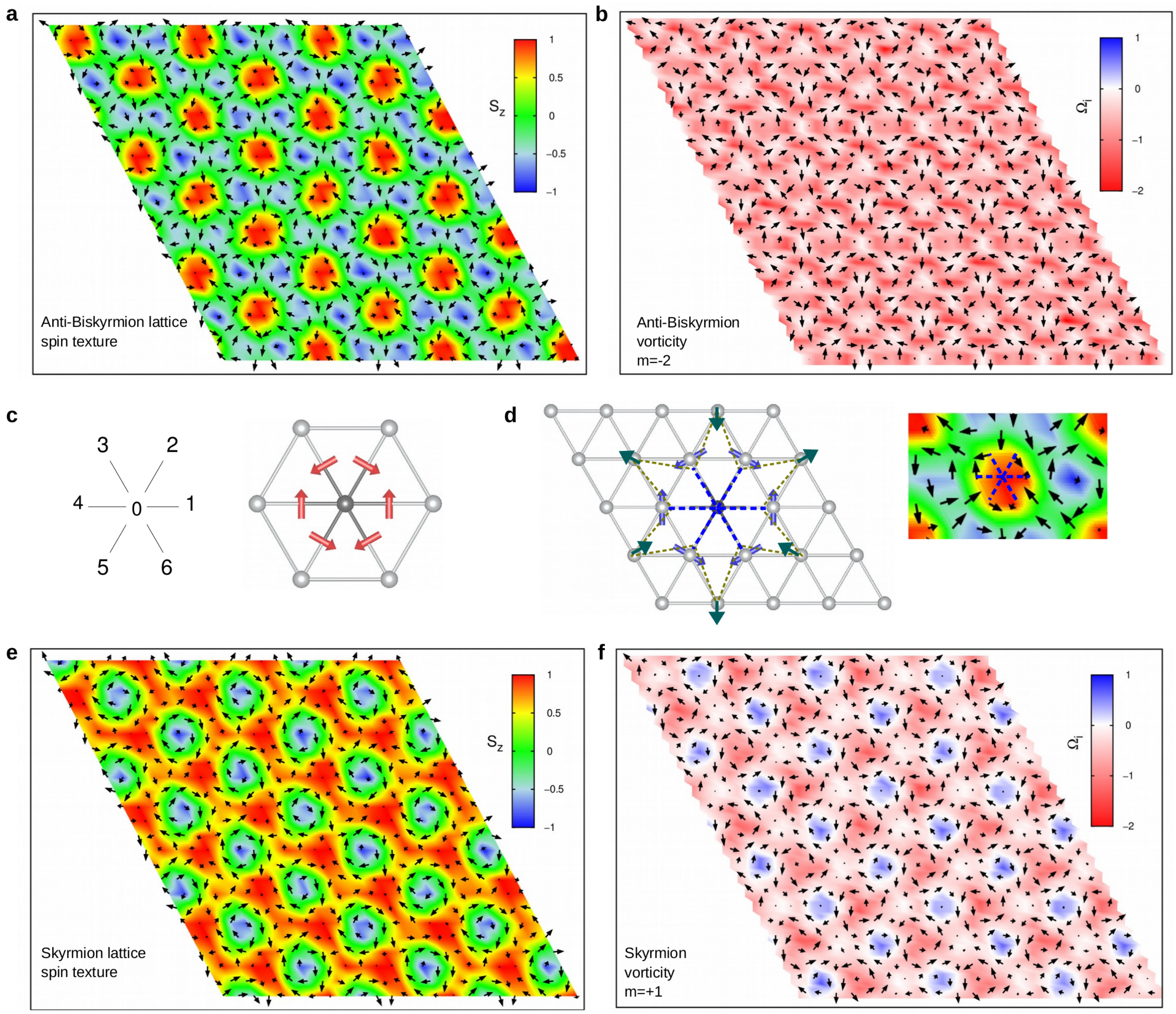

FIG. S3: Spin structures and topological charge of the thermodinamically stable phases in monolayer NiI$_2$. **(a,b)**, Snapshot from Monte Carlo (MC) simulations of the real space spin configurations and topological charge density map of the stable anti-biskyrmion lattice with $m = -2, \eta = \pi/2$ obtained for $B_z/J^{1iso} = 0$ on a 24×24 supercell with periodic boundary condition. Black arrows represent in-plane components of spins, the colormap indicates the out-of-plane spin component in **(a)** and the local scalar chirality in **(b)**. **(c)** In-plane projection for each Ni$_0$-Ni$_i$ pair of the non-coplanar $\boldsymbol{\nu}_\alpha$ eigenvector. The same labeling of Ni-pairs introduced in Fig. S1 is adopted. **(d)** Schematic representation of the spin configuration in the topological core: blue arrows represent spins on the nearest-neighbour Ni atoms which surround a central Ni$_0$; green arrows represent spins on the next-nearest neighbours with respect to Ni$_0$. Zoom on the anti-biskyrmion lattice helps visualization of the correspondence with the reconstructed spin pattern: spins on adjacent magnetic sites surrounding a central Ni$_0$ orient following the symmetric anisotropic interactions. **(e,f)** Snapshot from MC simulations of the real space spin configurations and topological charge of the skyrmion lattice with $m = 1, \eta = -\pi/2$ for $B_z/J^{1iso} \simeq 1.5$. **Note**: the purpose of this figure is to summarize the main findings reported in the main text, thus easing the comparison with the supporting results reported in Figs. S4 and S5.

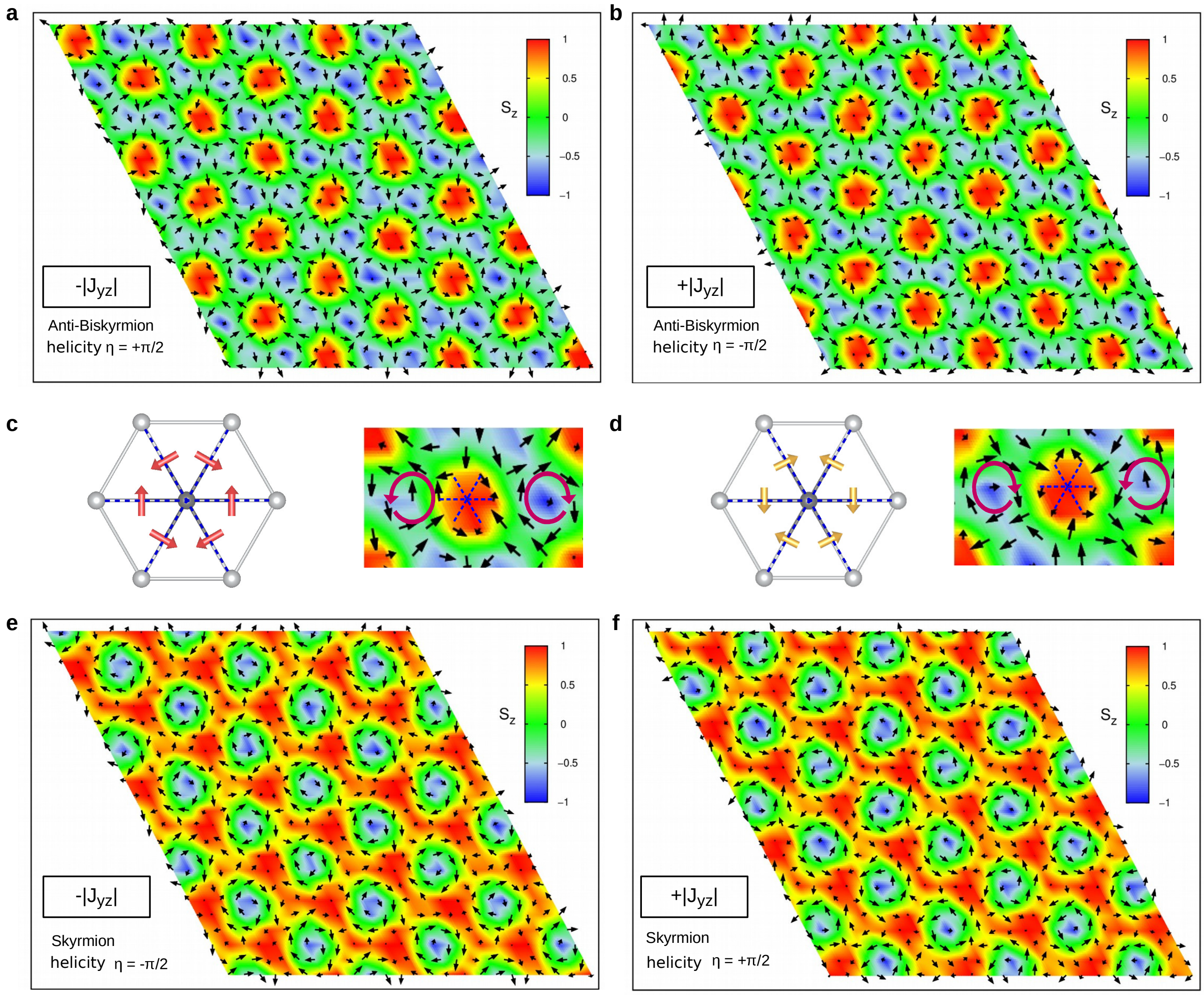

FIG. S4: Dependence of chirality on the sign of the off-diagonal component J_{yz}. **(a,b)** Snapshot from MC simulations of the real space spin configurations for monolayer NiI_2 obtained from DFT-calculated first- and third-nearest neighbours exchange interaction reported in the main text (Table I), keeping the negative sign of the J_{yz} terms in the Ni_0-Ni_1 pair **(a)** and changing its sign **(b)**. Change in the rotational sense of spins, *i.e.* the chirality of the anti-biskyrmion, is observed. **(c,d)** In-plane projection for each Ni_0-Ni_i pair of the non-coplanar $\boldsymbol{\nu}_\alpha$ eigenvector from the original calculated first-neighbor exchange interaction with negative J_{yz} (c), and from the first-neighbor exchange tensor where J_{yz} is imposed artificially to be positive (d). The applied change produces inversion in the (x, y) components of the eigenvector, that corresponds to reflection with respect to the {x,y} plane of an axial vector. Spins on the nearest-neighbour Ni atoms surrounding the central magnetic site of the topological core orient following the magnetic interaction, producing then the change of spins rotational sense also for the next-nearest neighbours, as highlighted in the zoomed view of the obtained spin configuration. A change of the chirality tuned by the sign of the J_{yz} term is also observed in the skyrmion lattice spin-texture obtained under an external magnetic field ($B_z/J^{1iso} \simeq 1.3$) **(e,f)**: helicity changes from $\eta = -\pi/2$ (e) to $\eta = \pi/2$ (f).

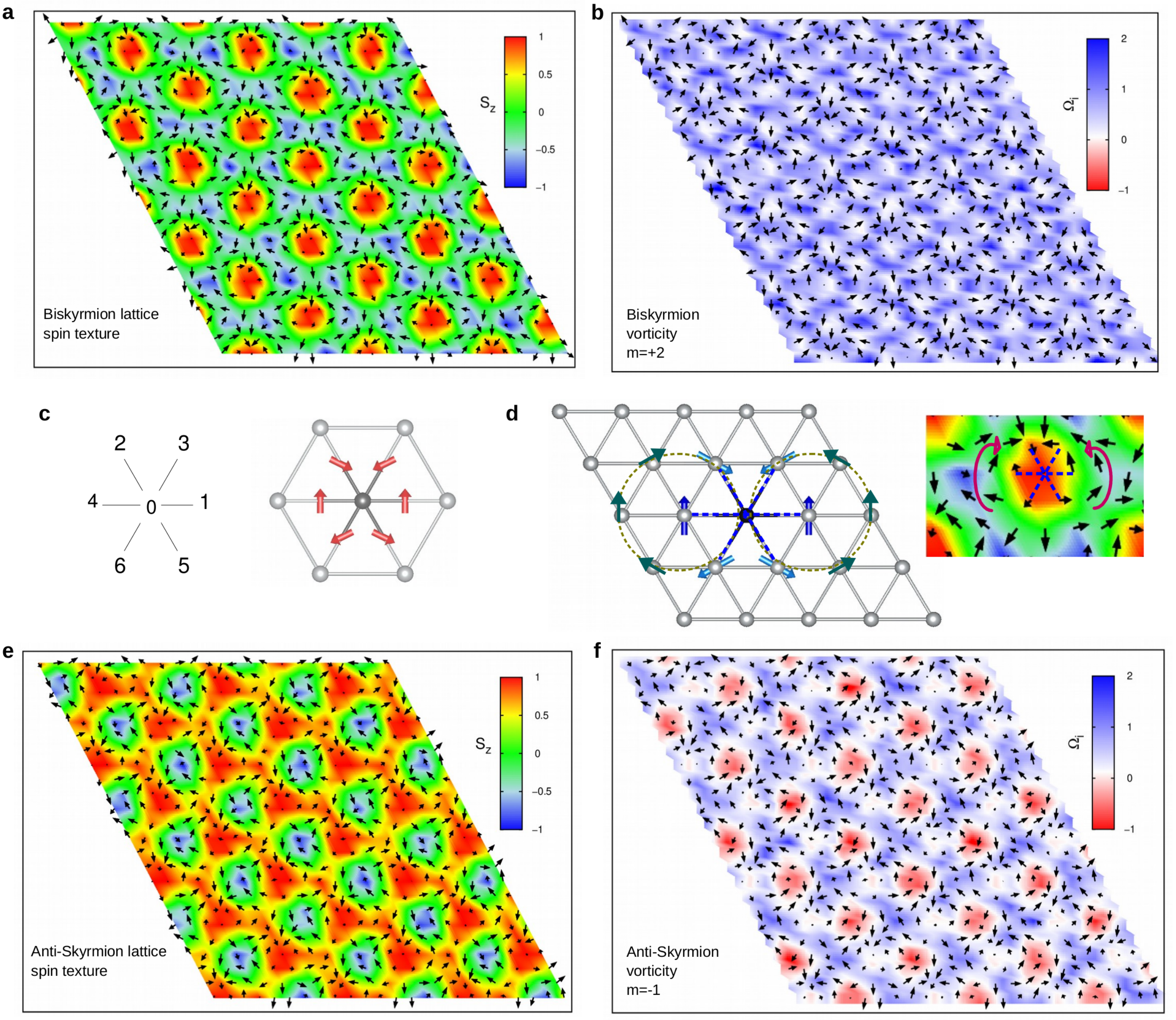

FIG. S5: Dependence of vorticity on two-site anisotropy in the triangular lattice. **(a,b)** Snapshot from MC simulations of the real space spin configurations and topological charge for monolayer NiI_2, as obtained from MC simulations with artificially modified nearest-neighbour anisotropic symmetric exchange: in closer detail, the two-site anisotropy terms have been swapped between Ni_0-$Ni_{2(5)}$ and Ni_0-$Ni_{3(6)}$ pairs, as shown in **(c)**. The resulting spin-texture shown in **(a)** corresponds to a bi-skyrmion lattice, with vorticity $m = 2$ as opposed to the spontaneous anti-bi-skyrmion lattice with $m = -2$. Accordingly, the spin configuration of the magnetic vortices accompanying the A2Sk-topological core also changes from "half"-skyrmion-wise to "half"-antyskirmion-wise. **(c)** In-plane projection for each Ni_0-Ni_i pair of the non-coplanar $\boldsymbol{\nu}_\alpha$ eigenvector related to the artificially set nearest-neighbour exchange interaction. **(d)** Reconstructed spin pattern from the magnetic exchange: blue (green) arrows represent spins on the nearest-neighbour (next-nearest neighbour) Ni atoms which surround a central Ni_0, compared with a zoomed view of the bi-skyrmion lattice. Spins on adjacents magnetic sites surrounding a central Ni_0 orient following the symmetric anisotropic interactions. **(e,f)** Anti-skyrmion lattice spin-texture and associated topological charge as obtained from MC simulation for the modified exchange tensor under an applied external magnetic field ($B_z/J^{1iso} \simeq 1.3$). Noteworthy, the obtained spin configuration displays an opposite vorticity $m = -1$ with respect to the skyrmion lattice ($m = +1$) found in the original, non-modified model. The results of these tests, alongside with the above examples reported in Fig. S3 and Fig. S4, confirm that the spin configuration (in particular its topology and chirality) is largely determined by the anisotropic symmetric exchange.

SIII. SUPPORTING MC AND DFT TESTS

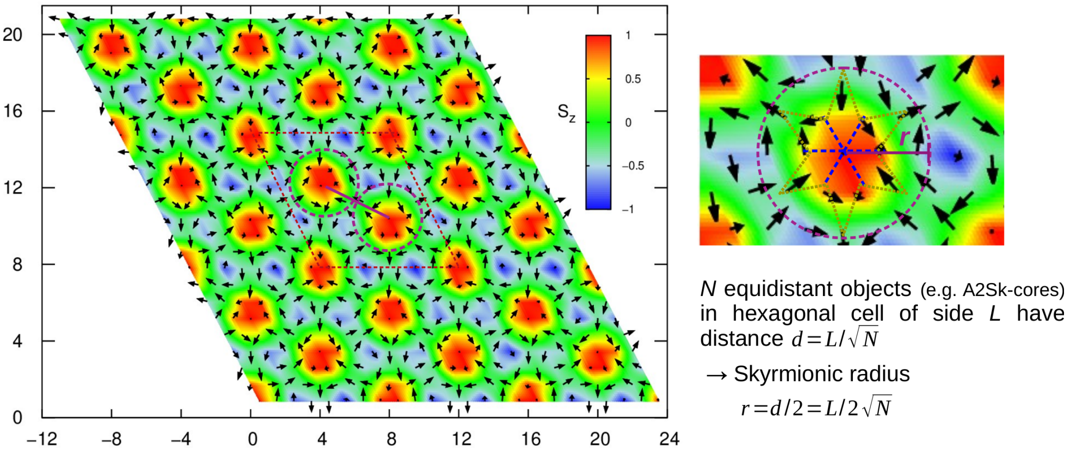

FIG. S6: Skyrmionic size. As further shown in next Fig. S7, the periodicity of the skyrmionic lattice and, then, the approximate size of the a single skyrmionic object are primarily determined by the strength of the magnetic frustration, *i.e.* the J^{3iso}/J^{1iso} ratio. From the predicted exchange interactions in monolayer NiI_2 reported in Table I of the main manuscript, we found that the minimal size L of the supercell, that is the magnetic unit cell ($m.u.c$), needed to accomodate the A2Sk-kattice is $\simeq 8a_0$, a_0 being the lattice constant of the unit cell. This value comes from the estimate of the propagation vector-$\mathbf{q}$ obtained by the evaluation of the spin structure factor $S(\mathbf{q})$ (Eq. 2 in the main-text) and it is also in agreement with the minimization of the isotropic exchange interaction in momentum space $J(\mathbf{q})$, given, e.g., in Ref [3]. In particular, the m.u.c. of the A2Sk lattice in NiI_2 (dashed lines inside the hexagonal 24x24 cell) comprises three anti-biskyrmions, each surrounded by six vortices. The number N of topological objects scales up with the size L of the magnetic supercell: $L \times L = nL_{m.u.c} \times nL_{m.u.c} = n^2(L_{m.u.c} \times L_{m.u.c})$; e.g. $24 \times 24 = 3^2(8 \times 8) \rightarrow N_{8\times8} = 3 \rightarrow N_{24\times24} = 3^2 \cdot 3 = 27$. The radius of each anti-biskyrmion can be then estimated as $r \sim L_{m.u.c}/2\sqrt{3}$; the A2Sk-diameter counts $\simeq$5 spins and thus it can be classified as atomic-scale skyrmionic structure [4, 5].

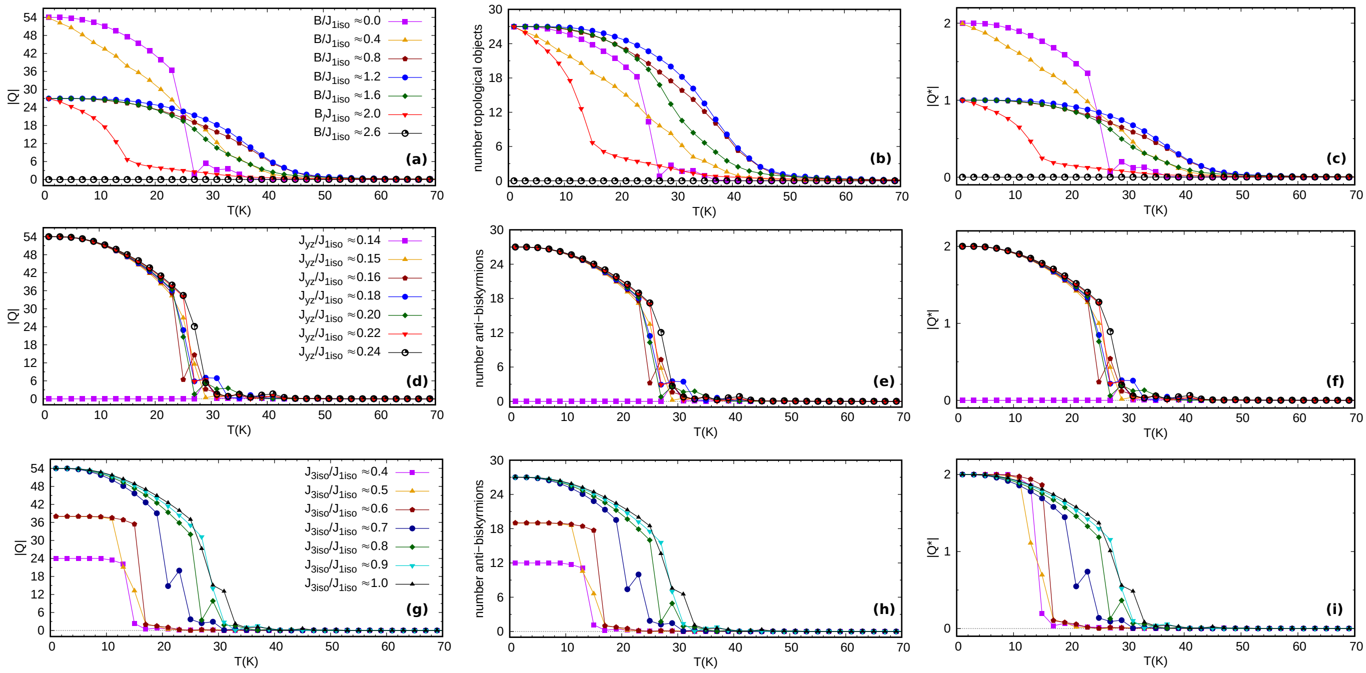

FIG. S7: Evolution of the topological properties as a function of the external magnetic field, the strength of the off-diagonal J_{yz} term and of the third-nearest neighbour (isotropic) interaction J^{3iso}. All parameters are expressed in units of the isotropic nearest-neighbour interaction J^{1iso}, the other interaction terms being kept fixed to the DFT-values obtained within the U=1.8 eV and J=0.8 eV approach for NiI_2 monolayer. Left panels column: absolute value of the total topological charge Q obtained from MC simulations on a 24×24 supercell. Central panel: number of topological objects (N) contained in the used supercell. Right panel: topological charge Q per magnetic topological object, obtained by normalizing the total charge with the number of topological objects ($|Q^*| = |Q|/N$). **(a,b,c)** Evolution with B_z. In agreement with the results shown in Fig. 1(f) of the main text, two sharp topological phase-transitions, signalled by the abrupt change of the total topological charge **(a)**, are induced under an applied magnetic field. Since the latter does not modify the periodicity - $L_{m.u.c.} \simeq 8$ with the given J^{3iso}/J^{1iso} - and hence the size of the magnetic unit cell, containing three magnetic cores, the number of topological objects in the ground state is always fixed to $3^2 \cdot 3 = 27$ **(b)**, while the topological charge of each magnetic bubble changes as $|Q^*|$ 2→1→0 with increasing applied field **(c)**. Transition temperatures are also dependent on the field strength. **(d,e,f)** Evolution with tuned J_{yz}. The total topological number, the number of cores - hence the size of each topological object - and the transition temperature are found to be overall robust against the strength of the off-diagonal anisotropic exchange; however, the topological A2Sk phase is not stabilized for $J_{yz}/J^{1iso} < 0.15$, where a topologically trivial single-**q** helimagnet state is stabilized, analogous to the one found in $NiBr_2$ (see Fig. 3 in the main text). **(g,h,i)** Evolution with tuned J^{3iso}. The change of total topological charge shown in **(g)** reflects the change of size of the topological objects, whose number is correspondingly reduced in the 24×24 supercell with decreasing J^{3iso}/J^{1iso} **(e)**, while the topological charge of each object remains fixed **(i)**. Indeed, the periodicity of the triple-**q** state is mostly determined by the ratio J^{3iso}/J^{1iso}; a smaller value of such ratio implies a larger magnetic unit cell, still hosting three topological cores. The largest biskyrmions are found for $J^{3iso}/J^{1iso} = 0.4$, with $L_{m.u.c} = 12$ and thus $N = 2^2 \cdot 3 = 12$ in the 24×24 simulation cell. We notice that similar size of the topological magnetic lattice ($L_{m.u.c} \sim 8$) is found in the range $0.7 \lesssim J^{3iso}/J^{1iso} \lesssim 1.0$, within which our DFT estimates for NiI_2 monolayer fall, consistently with the weaker dependence of the triple-**q** periodicity in this range.

	A_{Ni} (meV)
$NiCl_2$	0.0(0)
$NiBr_2$	0.0(1)
NiI_2	0.5(8)

TABLE SI: Single-ion anisotropy (SIA) calculated with U=1.8 eV, J=0.8 eV. We report here the coefficient A_i for a given nickel ion i of the SIA contribution to the magnetic interaction, which can be here simplified as $H_{SIA} = A_i S_{iz}^2$. Digits in brackets are below the accuracy of our DFT calculations (estimated to be within $\sim 10^{-4}$ eV). In fact, for NiI_2, we also verified that no further terms appear in the expansion of the single-ion anisotropy Hamiltonian [6]: coefficients A_{xy}, A_{xz}, A_{yz}, and $(A_{yy} - A_{xx})$ are in fact equal to zero; only the $(A_{zz} - A_{xx})$ is $\simeq +0.6$ meV, resulting thus into an easy-plane anisotropy. Moreover, we found that the SIA does not affect the spontaneous stabilization of the anti-biskyrmions lattice, being its contribution negligible with respect to the dominant J^{1iso} and J^{3iso} interactions and also with respect to the J_{yz} anisotropic term ($|A/J^{1iso}| \simeq 0.08$; $|A/J^{3iso}| \simeq 0.10$; $|A/J_{yz}| \simeq 0.4$). In fact, we performed MC calculations both without SIA and changing its sign, finding that the topology of the A2Sk and Sk lattices was unaffected.

	U=1 eV		U=2 eV		U=3 eV		U=1.8 J=0.8 eV	
	$\frac{J_{yz}}{J^{1iso}}$	$\frac{J^{3iso}}{J^{1iso}}$	$\frac{J_{yz}}{J^{1iso}}$	$\frac{J^{3iso}}{J^{1iso}}$	$\frac{J_{yz}}{J^{1iso}}$	$\frac{J^{3iso}}{J^{1iso}}$	$\frac{J_{yz}}{J^{1iso}}$	$\frac{J^{3iso}}{J^{1iso}}$
$NiCl_2$	0.00	-0.31	0.00	-0.31	0.00	-0.30	0.00	-0.33
$NiBr_2$	0.02	-0.47	0.02	-0.46	0.02	-0.50	0.02	-0.49
NiI_2	0.22	-0.84	0.20	-0.80	0.19	-0.79	0.20	-0.83

TABLE SII: J_{yz}/J^{1iso} and J^{3iso}/J^{1iso} estimated from $U = 1, 2, 3$ eV within the Dudarev approach and $U = 1.8$ $eV, J = 0.8$ eV within the Liechtenstein approach (reported in the main text). No substantial variation is observed in the ratio, thereby guaranteeing the realization of the anti-biskyrmion lattice in NiI_2 monolayer.

	J^{1iso}	J^{2iso}	J^{3iso}			
NiI_2	-6.7	-0.4	+7.1			
	$\mathbf{J}^{two-site\ aniso}$					
	J_{xx}	J_{yy}	J_{zz}	J_{yz}	J_{xz}	J_{xy}
NiI_2	-1.0	1.3	-0.3	-1.5	0.0	0.0

TABLE SIII: J^{1iso}, J^{2iso} and J^{3iso}, and two-site anisotropy ($\mathbf{J}^{two-site\ aniso}$) components (in meV) estimated for monolayer NiI_2 with lattice parameter fixed to the experimental one known for the bulk phase, *i.e.* $a = 3.89$ Å , within the the Liechtenstein approach ($U = 1.8$ $eV, J = 0.8$ eV).

[1] Xu, C., Feng, J., Xiang, H., and Bellaiche, L. Interplay between Kitaev interaction and single ion anisotropy in ferromagnetic CrI_3 and $CrGeTe_3$ monolayers. *npj Comput. Mater.* 4, 57 (2018).
[2] Xu, C., et al. Topological spin texture in Janus monolayers of the chromium trihalides $Cr(I,X)_3$. *Phys. Rev. B* **101**, 060404(R) (2020).
[3] Batista, C. D., Lin, S.-Z., Hayami, S.and Kamiya, Y. Frustration and chiral orderings in correlated electron systems. *Rep. Prog. Phys.* **79**, 084504 (2016).
[4] Nagaosa, N. and Tokura, Y. Topological properties and dynamics of magnetic skyrmions. *Nature Nanotechnology* 8, 899 (2013).
[5] Heinze, S., et al. Spontaneous atomic-scale magnetic skyrmion lattice in two dimensions. *Nat. Phys.* **7**, 713 (2011).
[6] Xiang, H., Lee, C., Koo H.-J., Gong, X. and Whangbo M.-H. Magnetic properties and energy-mapping analysis. *Dalton Trans.* 42, 823 (2013).
